# A Pilot Study of Polysubstance Use Sequences across the Lifespan among Assiniboine and Sioux People Who Use Injection Drugs

**DOI:** 10.3390/ijerph20010543

**Published:** 2022-12-29

**Authors:** Michael Anastario, Paula Firemoon, Ana Maria Rodriguez, Carrie Wade, Christopher Prokosch, Elizabeth Rink, Eric Wagner

**Affiliations:** 1 Robert Stempel College of Public Health & Social Work, Research Center in Minority Institutions, Florida International University, Miami, FL 33199, USA; 2 Fort Peck Community College, Poplar, MT 59255, USA; 3 Independent Researcher, Belize City, Belize; 4 University of Minnesota Medical School, Minneapolis, MN 55455, USA; 5 Department of Health and Human Development, Montana State University, Bozeman, MT 59717, USA

**Keywords:** injection drugs, polysubstance use, methamphetamine, hepatitis C, life history calendar, social sequence analysis, American Indians/Alaska Natives

## Abstract

Compared with other racial/ethnic groups in the United States, American Indians/Alaska Natives have the highest rates of acute Hepatitis C Virus (HCV) infection, the highest HCV-related mortality, and one of the fastest climbing rates of drug overdose deaths involving stimulants. In this pilot study, a life history calendar was administered to Indigenous people who use injection drugs (IPWIDs) to understand sequences of polysubstance use across the lifespan. 40 IPWIDs completed a questionnaire and life history calendar. Social sequence analysis was used to examine patterns in sequential phenomena among substances reported over years of the lifespan. Most participants (55%) began injecting substances before the age of 21, 62.5% shared syringes with others, and 45% had ever been diagnosed with HCV. An appreciably large increase in the use of stimulants occurred between the year prior to and following injection initiation (33% to 82%). A three-cluster solution distinguished younger IPWIDs transitioning into polysubstance use involving stimulants and/or narcotic analgesics from adults using narcotic analgesics with stimulants over longer periods of time, and adults most focused on stimulant use over time. Findings from this pilot study contribute to an understanding of how methamphetamine injection plays a role in the HCV epidemic among IPWIDs.

## 1. Introduction

Compared with other racial/ethnic groups, American Indians/Alaska Natives (AI/ANs) have the highest rates of acute HCV infection (3.6 reported cases per 100,000), HCV-related mortality (8.6 per 100,000), and show climbing rates of drug overdose deaths [1,2,3,4]. Indigenous people who use injection drugs (IPWIDs) experience elevated risks for both HCV infection and overdose, however little is known about substance use patterns that affect HCV risk within IPWIDs. In one study of Indian Health Services health care encounters, it was documented that 25.6% of adults aged 18–35 had concurrent diagnoses of HCV infection and opioid use disorder (OUD) [5]. This pilot study aimed to document patterns in polysubstance use across the lifespan among IPWIDs in a tribal community that experienced an influx of illicit substances due to the development of nearby oil drilling over the past decade. An improved understanding of polysubstance use across the lifespan among IPWIDs will inform future research and intervention designs that aim to reduce the risks of HCV and overdose among IPWIDs.

The alterity of IPWIDs reinforces their risk profile at the nexus of an emerging syndemic. In one study of US northern plains IPWIDs, over half of the sample reported using and injecting substances before the age of 24, the majority reported syringe reuse, and there was low knowledge of the causes of HCV, its modalities of transmission, and the course of the disease [6]. Economic disparities undermine access to antiviral drugs for AI/ANs, with Indian Health Services per capita user health expenditures being less than half that of the US general population [7,8]. In one qualitative study of IPWIDs in the US, IPWIDs described poor access to services across multiple sectors, experiences of felt stigma, human capital deficiencies among providers, and community overreliance on the criminal justice system to address myriad health and social service needs [9]. The social marginalization of IPWIDs reinforces a general opaqueness regarding strategies to mitigate IPWID health risks and excess disease burdens.

Researching patterns of substance use in IPWIDs is undermined by a historical tendency to analytically omit structural violence from an understanding of AI/AN substance use susceptibility [8]. Traumatic experiences with boarding school practices, the provision of poor social services, economic poverty, unemployment, multiple exposures to various forms of violence and early childhood adversity are implicated in substance use risk among AI/ANs [10,11,12,13,14,15,16,17]. Substance use research in tribal communities has often relied on western/settler approaches built on reductionist (vs. contextualist), biomedical (vs. spiritually and socially focused), problem-focused (vs. strengths-focused), and intrapsychic (vs. relational) approaches [18]. Despite attempts to incorporate “culture” into studies with Indigenous people, substance use research also continues to rely heavily on mainstream constructs and assessments (e.g., self-efficacy and the Addiction Severity Index). The validity of these instruments for Indigenous people remaining largely unknown [19,20] Western research instruments for substance use with Indigenous people have accomplished little to articulate with Indigenous methodologies such as storytelling and remembering [21], which may offer alternative ways of thinking about IPWID substance use patterns and related health outcomes such as HCV infection. More interactive and less linear methods of documenting substance use over the lifespan that are conducive to the elicitation of stories may provide a more culturally appropriate experience of research in some populations, particularly those with heightened sensitivity to interrogation and stigma. 

The development of instruments that assign a quantitative value to IPWIDs’ unique ways of explaining and remembering polysubstance use across the lifespan benefit from understanding the temporal and spatial contexts in which those stories are told. Engagements with shifting opioid medications in tribal communities over time may be one factor that contributes to injection initiation and ongoing opioid use among IPWIDs. Historically, opioid medications in the US have evolved from short-acting to extended-release and abuse-deterrent formulations over the past 50 years [22,23,24]. One birth cohort study of non-Indigenous people who use injection drugs (PWIDs) found shorter transition times to injection among participants whose entry into adolescence and adulthood overlapped with the emergence of prescription opiate drugs in the 1990′s and 2000′s [22]. In another study of PWIDs in the US, oxycodone/acetaminophen or long-acting oxycodone use preceded a transition to heroin, which usually began with nasal inhalation or smoking, followed by a transition to injection drug use [25]. While prescription opioid availability and misuse may increase the likelihood of injection initiation among individuals who use substances, subsequent substance use patterns may in turn affect HCV risk in PWIDs. For example, the preparation of cool water-soluble heroin for injection requires different drug heating processes than ‘black tar’ heroin, potentially resulting in differential infectious disease transmission risks as a result of how the substance is heated prior to injection [26,27,28]. The more recent historical development of agonist (Methadone) and partial agonist (Buprenorphine/Naloxone) treatments for OUD has also produced the under-researched phenomenon of using diverted medication for OUD (MOUD) [9,29,30]. Shifting opioid formulations are not the only documented drivers of transitioning to injection drug use among PWIDs, as factors such as pre-existing substance use, social networks, the mismanagement of pain, and curiosity have been documented as contributing factors [31,32,33,34]. However, the development and increasing availability of potent opioid medications on or near tribal lands raise questions about how these medications are used relative to injection initiation among IPWIDs.

The growth of the opioid epidemic in the US has given way to an opioid-centric perspective that eschews how the majority of people with a substance use disorder engage in polysubstance use [35,36]. Several studies have found that opioid use co-occurs with at minimum one nonopioid substance, and there is increasing evidence of polysubstance use involving methamphetamine and opioids [37,38,39]. In one nationally representative study of patients in drug treatment programs across the US, an increase in the past month’s use of methamphetamine in individuals was observed among patients with opioid use disorder from 2011 to 2017 [40]. In another study of care-engaged individuals who use methamphetamine in an urban setting, the vast majority had opioid use disorder (94%) and were being treated with medications for opioid use disorder, but none were receiving contingency management for methamphetamine use disorder [41]. Polysubstance use and poly-route use involving stimulants and opioids have been documented in PWIDs [42,43] and may be associated with increased risks for injection-related risk of blood-borne pathogen acquisition [44].

Spatially, the flow of illicit substances into tribal communities is partially driven by neocolonial phenomena in the US, including the incursion of settler extractive industrial activity on or near tribal lands. The recent development of the Bakken oil formation, which has its epicenter in North Dakota (US), but which also spans Montana (US) and Saskatchewan (Canada) has led to unprecedented population growth in the region, criminal activity, and drug trafficking [45,46]. This region is home to multiple tribal communities that include Assiniboine, Sioux, Mandan, Hidatsa, Arikara, Blackfeet, and Gros Ventre people. From 2008 to 2022, the number of wells producing oil in North Dakota has more than quadrupled [47], which has led to an influx of labor associated with the oil boom. The development of oil drilling operations in North Dakota has coincided with a rise in the number of drug overdose deaths [48]. The development of the Bakken oil formation has resulted in the circulation of illicit fentanyl and methamphetamine through tribal territories located along the Hi-Line region surrounding US Highway 2 in Montana, a transportation route that links directly to the hub of drilling operations in Western North Dakota [49]. The unintended public health consequences of the economic development of the Bakken oil formation carry direct implications for health issues such as HCV and abscesses among IPWIDs who lack reliable access to harm reduction services. During the oil boom period, one local syringe exchange program was implemented through the Tribal Health department, providing access to syringes, sharps containers, tourniquets, alcohol pads, and condoms. Syringes were exchanged until 2020 despite an ongoing need for syringe exchange. A study conducted with IPWIDs in the region during the period that the syringe exchange program existed identified that nearly a quarter of IPWIDs still reported that it was difficult to obtain new syringes [6]. Since the discontinuation of the program in Fort Peck, the nearest syringe exchange program for IPWIDs is located three hours away on the Fort Belknap Reservation, and there is limited naloxone awareness and access in the region. The high rates of overdose among individuals in the sustained opioid use cluster are particularly concerning in this context. The transportation required to access the program makes it an infeasible option for IPWIDs living on the Fort Peck Reservation to reliably access syringes.

Thinking about the development of instruments to assess substance use and injection patterns across the lifespan of IPWIDs is challenging due to concerns regarding cultural appropriateness, and the increasing recognition that syndemic assemblages are local and heterogeneous [50,51]. Life history calendars (LHCs) allow for the collection of quantitative data in a visual, grid-format timeline comprised of multiple domains over a defined time period that serves as an alternative format to close-ended questionnaires and timelines. LHCs may improve recall ability and data quality for respondents in an interview setting [52,53], and have been used in a variety of substance use-related studies that include alcohol, marijuana, opioids and other injection drugs [54,55,56,57]. In one study of adult opioid users, the LHC was found to provide rich data regarding life events, living situations, personal experience, and developmental periods relevant to the individual’s substance use experience [54]. By capturing shared experiences of substance use that emerge through synchronous acts (e.g., injecting similar substances over time) and eventually shape social roles (e.g., distinguishing groups with unique substance use patterns and treatment needs), the analysis of LHCs may offer one possibility to accommodate nonlinear stories told by participants who wish to explain their substance use, while simultaneously being a method that can translate sequences of substance use into quantitative values informative to Western scientific endeavors.

In this study, we piloted a life history calendar (LHC) with Fort Peck Tribal members who use injection drugs living near the development of the Bakken oil formation. The aims of this study were to explore the validity of lifetime substance use measures and to examine polysubstance use sequences and HCV risk over the lifespan. Findings have direct implications for understanding and responding to the complex health needs of IPWIDs living in this region.

## 2. Materials and Methods

### 2.1. Setting and Study Design

The study took place at Fort Peck Community College (FPCC) on the Fort Peck Reservation in northeastern Montana. Approximately 8000 enrolled tribal members, predominately from the Sioux and Assiniboine Nations, live on the 2.1-million-acre reservation space. The Sioux comprise Sisseton/Wahpetons, Yanktonais, and Teton Hunkpapa bands, and the Assiniboine comprise Wadopana and Hudashana bands [58]. This current study builds on ongoing community-based participatory research (CBPR) between members of the study team and the Fort Peck tribes over the past 15 years. 

The original plan to recruit IPWIDs was to use chain-referral sampling, which begins with selecting seed participants (one male, one female) and proceeds with study participants recruiting other IPWIDs through word of mouth and invitation by the previous participant. This sampling strategy was chosen given that members of the target population know one another and are densely interconnected [59,60]. For this study, the coupons used for the chain-referral sampling approach were eventually deemed not useful given that word-of-mouth references spread faster than the study coupons, and the majority of participants arrived at the study site without a coupon. Participants were screened for eligibility by the study’s tribal director prior to participating in the interview. 

To be eligible for the study, participants had to meet each of the following inclusion criteria: (1) Be currently injecting drugs or previously injected substances for ≥12 months; (2) Be a registered member of a federally recognized tribe or an associate tribal member; and (3) Be greater than 13 years of age. The study’s only exclusion criterion was being currently incarcerated and/or in police custody at the time of the study. All participants provided verbal consent prior to participating in the study. All study participants received a $50 gift card for participating in the interview. Human Subjects approval was obtained from the Fort Peck Institutional Review Board (IRB) and the Florida International University IRB (Protocol Approval # IRB-21-0177). 

Three interviewers verbally administered a questionnaire and LHC. LHCs were administered on “30 × 42” poster-sized sheets of paper or digitally on a computer screen that was visible to the participant. On average, the interview took 47 min to complete (ranging from 25 to 90 min).

### 2.2. Measures

Demographic characteristics. The questionnaire included self-reported gender identity, age, year of birth, relationship status, tribal registration or associate tribal member status, and tribal identification (Assiniboine, Sioux, both, other). Associate tribal members are individuals of one-eighth or more but less than one-quarter, Assiniboine and/or Sioux blood born to any member of the Assiniboine and Sioux Tribes, provided they are a citizen of the United States at the time of birth.

Injection-related health outcomes. The questionnaire included questions regarding who first taught the participant to inject, whether the IPWID had ever taught anyone else to inject (no, yes), and how frequently syringes were currently shared with others (1 = never, 2 = sometimes, 3 = always). The questionnaire included one open-ended question about the first injection event: “why did you decide to inject?” Responses were subject to an inductive coding strategy that produced four categories: 1. curiosity, 2. intensity of the high, 3. influence in peer networks, or 4. coping with a stressful life event. One open-ended question about the participant’s typical injection routine was subsequently coded for whether the participant regularly used any type of filter (cotton, cigarette butts, napkins, tissue) during the injection process (0 = no filter used, 1 = filter used). The LHC also contained items regarding years of life in which the participant experienced abscesses and overdose, as well as whether a healthcare provider had ever diagnosed the participant with Hepatitis C, HIV, any other infectious disease, OUD, addiction or dependency diagnosis, diabetes, hypertension, heart disease, liver disease, cancer, or another illness during their lifetime. Attempts to obtain medication for OUD during a given year of life were also documented on the LHC.

Lifetime polysubstance use via the ASI. To evaluate the validity of the LHC, the drug/alcohol use module of the ASI 5th Edition adapted for use with American Indians [61] was included in the questionnaire (administered to participants prior to the LHC) to measure substance use over the lifespan. Polysubstance use was classified as using more than one substance during an overlapping 1-year timeframe. While this timescale cannot establish the concomitant use of two substances on one occasion, it does illustrate polysubstance use within a given year. The module screens participants for use of alcohol, heroin, methadone, other opiates/analgesics, barbiturates, sedatives/hypnotics/tranquilizers, cocaine, amphetamines, cannabis, hallucinogens, inhalants, and more than one substance used per day. For each substance, participants were asked whether they had ever used the substance in their lifetime (yes/no), whether they had used the substance in the past 30 days (yes/no), age at first use, years of use, route of administration, and age of last use. The instrument also screens participants for the traditionally sanctioned use of substances, whether traditional Indian cultural practices (e.g., sweat lodges, sun dances, prayer meetings) have been helpful in achieving abstinence, overdose events, and treatment for alcohol and drug abuse. For the purposes of this study, ever having used a substance (0 = never, 1 = at least once) and the number of years of reported use for each substance was calculated.

Lifetime polysubstance use via the LHC. After completing the ASI, LHC forms were used to measure substance use over the lifespan. Individuals were first asked to explain where they had lived since birth, followed by the year the participant began injecting substances, and the individual parts of the body (arms, legs, neck, groin, fingers/toes, other) that the participant had injected substances into during a given year of life. After establishing this information to serve as potential anchors, an inventory of substances used was plotted, by year, over the lifespan. Polysubstance use was classified as using more than one substance during an overlapping 1-year timeframe. The items in the substance use inventory were based on substances previously described in formative qualitative research with IPWIDs [9]. Participants were asked to indicate the years of life they used: Alcohol, methadone, Suboxone^®^, buprenorphine, heroin, other opiates/analgesics, barbiturates, sedatives/hypnotics/tranquilizers, cocaine, amphetamines, methamphetamines, cannabis, hallucinogens, inhalants, speedballs (heroin and cocaine mixed together), goofballs (heroin and speed mixed together), injected energy drinks, injected tap water, injected saline solution, and other substances. Other substances reported included varying definitions of speedballs (typically, variations of opioids and methamphetamine). To compare results with the ASI, ever having used a substance (0 = never, 1 = at least once) and the number of years of reported use were calculated for each individual substance derived from the LHC. The number of years of reported use was calculated by summing the cumulative number of years where a substance was documented (using horizontal lines on the LHC indicating a year in which the substance was used).

Polysubstance use combinations. Polysubstance use combinations (PUCs) were developed across individual substances reported during any given year of life on the LHC to inform the social sequence analyses. As a nominal variable, raw PUCs included 106 distinct combinations. To reduce the variability of the metric for use in the social sequence analysis, substances were grouped into Drug Recognition Expert (DRE) categories (CNS depressants, CNS stimulants, hallucinogens, narcotic analgesics, inhalants, and cannabis) [62]. All possible combinations of each DRE category were calculated for each person-year, producing a nominal variable containing 30 PUCs for any year of life across the 40 IPWIDs in the sample. To further reduce variability in the metric, DRE category combinations observed at a frequency of *n* ≤ 4 in the unbalanced panel dataset of 1513 observations (<0.38%) were combined with the nearest PUC (e.g., the low-frequency depressant/narcotic analgesics/cannabis/hallucinogen PUC would be reclassified as belonging to the higher frequency depressant/narcotic analgesics/cannabis PUC). This final variable included 19 PUCs that were used to develop the social sequence analysis. 

### 2.3. Data Analysis

The data analysis plan included univariate analyses (examination of frequency distributions and measures of central tendency/dispersion), bivariate tests comparing substance use between the ASI and LHC, and social sequence analysis. All analyses were conducted using STATA 14.

To compare individual substances reported between the ASI and LHC, McNemar’s chi-square test was used to evaluate marginal homogeneity in the detection of the substance, and Pearson correlation coefficients were used to compare years of reported use. To control for Type I errors due to multiple tests, a Bonferroni-Holm correction procedure was used to determine statistical significance; beginning with *p* values < 0.00238 (0.05/21) and continuing until the *i*th ordered *p* value was p(i) ≥ α/(K − i + 1), at which point the value was not considered to be statistically significant [63,64].

Social sequence analysis was used to examine patterns in sequential phenomena among substances reported over years of the lifespan. This included developing probability state transition matrices (PSTMs) from the unbalanced panel data (*n* = 1513 person-years) to determine whether a given substance immediately preceded or immediately followed the year of injection drug use initiation. PSTMs are useful in illustrating whether there are adjacent and/or systematic relationships between pairs of elements in substance use sequences [65]. A PSTM is a square matrix containing *k × k* cells:

(1) $$P=\left(\begin{array}{ccc} p\left(\mathrm{AA}\right) & \cdots & p\left(\mathrm{AK}\right)\\ \vdots & \ddots & \vdots \\ p\left(\mathrm{KA}\right) & \cdots & p\left(\mathrm{KK}\right) \end{array}\right)$$

where k is the number of elements (substances/first injection events) in the element universe, P_AK_ is the probability that substance A is followed by injection initiation year K, P_KA_ is the probability that the year of injection initiation is followed by substance A, and where *p*(AK) = *n*(AK)/*n*(A). Panel data were subsequently balanced by including observations from all participants 20 years prior to the date of the interview (*n* = 800 person years). Optimal matching was conducted using SQ-Ados in Stata [66,67]. Indels and substitutions were used to align sequences, and alignment costs were weighed using the Levenshtein distance of 1 for each. A square dissimilarity matrix containing the Levenshtein distances was used to develop an agglomerative hierarchical cluster analysis. Visual inspection of dendrograms was used to evaluate the number of classes [65]. Other class membership criteria were considered, but caution was exercised given a large number of PUCs relative to the small number of participants. Sequence index plots were used to aid in the visual interpretation of sequence cluster analysis findings. Finally, we explored whether the different clusters that emerged were similar or different regarding injection-related outcomes (overdose, number of times attempted to obtain MOUD during the lifespan, treatment for drugs or alcohol, and HCV infection). For these exploratory analyses, statistical significance was not considered as these analyses assist in characterizing the clusters.

## 3. Results

### 3.1. Participant Characteristics 

Twenty-two (55.0%) participants self-identified as male, and 18 self-identified a female (45.0%). Most participants (72.5%) were 30–50 years of age. Twenty-two participants reported a relationship status of single (55.0%), followed by unmarried with a partner (25.0%), married (10.0%), widowed (5.0%) and divorced/separated (5.0%). Thirty-five participants (87.5%) were registered members of a federally recognized tribe, and five (12.5%) were associate tribal members. For tribal affiliation, twenty-four participants (60.0%) identified as Sioux, four as Assiniboine (10.0%), nine as both Assiniboine and Sioux (22.5%), and three as Other (Chippewa, Innuit, Potawatomi) (7.5%) (Table 1). 

### 3.2. Injection Drug Use and General Health Characteristics

Slightly more than half of the participants (55%) began injecting substances before the age of 21. Most learned to inject substances from family members (32.5%), followed by friends (25.0%) and romantic partners (17.5%). Reasons for first injecting substances included curiosity (30.0%), the intensity of the high (25.0%), peer influence (35.0%), and coping with a stressful life event (10.0%). Several participants (27.5%) had taught someone else to inject substances. Most participants shared syringes with others (62.5%). Only 22 (56.4%) mentioned using a filter for injection preparations prior to drawing the substance into the barrel of the syringe (Table 2). Nine participants (22.5%) reported ever having experienced an overdose, and 22.5% reported an abscess at an injection site on the body. Eighteen participants (45%) reported ever being diagnosed by a health professional with Hepatitis C. When asked what services they would like to see for IPWIDs, 60% reported syringe exchange.

### 3.3. Lifetime Recall of Substances Used via the ASI and LHC

No statistically significant differences were observed in the detection of any lifetime substance use between the ASI and LHC. However, the LHC explicitly screened participants for methamphetamine use (95.0%) apart from amphetamine use (37.5%). The ASI combines methamphetamine use with amphetamine use, which received 57.5% endorsement. Fifteen IPWIDs who reported any lifetime use of methamphetamine on the LHC were not detected on the ASI amphetamine screener. The LHC also screened for the use of diverted Suboxone^®^, which was endorsed at 42.5%.

The greatest similarities in the years of reported use between the ASI and LHC occurred for inhalants (r = 0.84, *p* < 0.001), alcohol (r = 0.82, *p* < 0.001), and cannabis (r = 0.68, *p* < 0.001). The greatest divergence was observed for methadone (r = 0.01), hallucinogens (r = 0.17) and cocaine (r = 0.19) (Table 3). 

### 3.4. Social Sequence Analyses (Table 4)

PTSM results are illustrated in Table 4 (columns 4 and 6). In the year immediately prior to injection initiation, PUC transition probabilities were 0.13 for using depressants/narcotics analgesics, 0.11 for using depressants/hallucinogens/cannabis, 0.10 for depressants alone, 0.09 for depressants/inhalants/cannabis, and 0.08 for depressants/stimulants/narcotic analgesics/inhalants/cannabis (Table 4). During their first year of injection drug use, most participants (70%) had a PUC involving a minimum of depressants/stimulants/cannabis (Table 4). In the year immediately following injection initiation, PUC transition probabilities were 0.33 for depressants/stimulants/cannabis, and 0.23 for depressants/stimulants/narcotic analgesics/cannabis (Table 4). Across all PUCs, an appreciably large increase in the use of stimulants occurred between the year prior to and following injection initiation (33% to 82%). The proportion of person-years where the respondent had a Hepatitis C diagnosis by a healthcare professional within a given PUC for that same period is shown in the third column of Table 4. Hepatitis C was most prevalent for PUCs involving stimulants/narcotic analgesics (94%) and stimulants/narcotic analgesics/cannabis (46%) (Table 4).

**Table 4:** Probability state transition values for substance use sequences relative to first injection event in an unbalanced panel of Assiniboine and Sioux people who use injection drugs, *n* = 1513 person-years for 40 participants.

Polysubstance Use Combination (PUC)	Total Number of Person-Years	Proportion of Person-Years in PUC (Row) with Hepatitis C	Probability That PUC Precedes First Year Ever Injecting Drugs **	Number of Participants with PUC during the First Year of Drug Injection	Probability That PUC Immediately Follows the First Year of Injecting Drugs **
c	75	0.00	0.05		0.00
d	31	0.00	0.10		0.03
d + c	237	0.00	0.06	1	0.10
d + h + c	9	0.00	0.11		0.00
d + i + c	12	0.00	0.09		0.00
d + n	8	0.00	0.13		0.00
d + n + c	20	0.00	0.06	1	0.05
d + s	72	0.04	0.00	3	0.08
d + s + c *	282	0.24	0.02	12	0.33
d + s + h + c *	23	0.00	0.05	4	0.05
d + s + n	41	0.02	0.05	3	0.05
d + s + n + c *	100	0.08	0.05	10	0.23
d + s + n + i + c *	16	0.06	0.08	2	0.00
i	4	0.00	0.00		0.00
s	21	0.05	0.06	1	0.03
s + c	62	0.00	0.02	3	0.05
s + n	16	0.94	0.00		0.00
s + n + c	13	0.46	0.00		0.00
None	471	0.01	0.00		0.00

Abbreviations: C Cannabis; D Depressants; H Hallucinogens; N Narcotic Analgesics; S Stimulants; I Inhalants; PUC Polysubstance use combination. * PUC variations that include a combination of CNS depressants, stimulants, and cannabis. ** Probability State Transition Matrix values derived from the unbalanced panel data.

Following optimal matching, a three-cluster solution was identified. The solution is presented graphically through sequence index plots for each cluster in the Supplementary Materials. The clusters were interpreted as differentiating IPWIDs who were:

1. Younger and just transitioning into polysubstance use involving stimulants and/or narcotic analgesics (*n* = 10),
2. Adults engaged with narcotic analgesics and stimulants over longer periods of time (*n* = 16), and
3. Adults most focused on stimulant use over time (*n* = 14).

The exploratory analysis revealed that individuals in cluster 2 had on average 2.1 (SD = 6.9) overdose experiences, which was more than individuals in cluster 1 (mean = 0.3, SD = 0.7) and cluster 3 (mean = 0.2, SD = 0.6). Individuals in cluster 2 also attempted to obtain MOUD more frequently during their life (6.3%) in comparison to individuals in cluster 1 (0.5%) or cluster 3 (1.1%). The number of times treated for alcohol or drug abuse was similar across cluster 1 (mean = 1.8, SD = 2.4), cluster 2 (mean = 2.0, SD = 1.3), and cluster 3 (mean = 2.1, SD = 2.4). The self-reported prevalence of Hepatitis C was highest in cluster 3 (50.0%), followed by cluster 2 (37.5%) and cluster 1 (20.0%).

## 4. Discussion

In this pilot study of 40 IPWIDs living in northeastern Montana, we aimed to explore the validity of lifetime substance use measures and to examine polysubstance use sequences and HCV risk over the lifespan. Our use of LHCs provided some minor benefits in assisting participants with recall concerning specific phases of life during which substances were used. In the sample, we identified high rates of self-reported HCV infection, increases in stimulant use surrounding injection initiation periods, and within-group heterogeneity in sequences of engagement with narcotic analgesics (mainly opioids) and CNS stimulants (mainly methamphetamine) over the lifespan. Results inform an understanding of multiple concurrent harms associated with injection drug use practices among IPWIDs in the syndemic context.

This study expands an understanding of the HCV/OUD syndemic among AI/ANs by showing relatively high rates of methamphetamine use coupled with HCV infection among northern plains IPWIDs. In this community sample, we observed an appreciably large increase in stimulant use immediately surrounding the year of injection initiation, which could indicate a rapid progression from smoking/hot-railing methamphetamine to injecting methamphetamine. Social sequence analysis findings suggested the existence of differential trajectories of polysubstance use involving stimulants over the lifespan. While a relatively smaller cluster of IPWIDs reported sustained opioid use (often in combination with methamphetamine), the majority of participants were most focused on methamphetamine use over the life course. IPWIDs in the cluster with sustained opioid use showed higher rates of overdose and attempts to obtain MOUD during the life course, whereas individuals in the methamphetamine focused cluster showed the highest rates of self-reported HCV. An increase in deaths from opioids in combination with methamphetamine and other simulants has been observed in minority populations, nationally [68]. Findings are also consistent with AI/ANs being the racial/ethnic group showing the largest recent rate increase and highest death rate in stimulant-involved death rates in comparison to other racial/ethnic groups [69].

The results concerning polysubstance use sequences suggest differential treatment and service needs within IPWIDs, some of whom are more engaged with opioids over a longer period in comparison to others whose injection use centers exclusively on methamphetamines. While MOUD and naloxone access need to be addressed within IPWIDs showing sustained opioid use, particularly given the high rates of overdose among IPWIDs in cluster 2, there are no community-based stimulant overdose response medications/treatments that health professionals can use to address stimulant-involved overdoses. This latter point reflects a broader lack of research and development concerning simulant-involved overdose. Within-group heterogeneity in polysubstance use sequences across the lifespan may warrant varying and targeted response strategies to address differential HCV risks within different clusters of polysubstance use. Many of these HCV risks could be directly addressed by re-implementing a local syringe exchange program.

The study also identified additional health risks faced by IPWIDs in this community. Dissolving methamphetamine inside the syringe barrel without filtration nor heating was a common technique described among participants, and in part reflects the lack of a local harm reduction program that could otherwise provide reliable access to drug injection equipment. This particular injection practice that eliminates heating and filtration of the injection preparation may contribute to and/or aggravate excess infectious disease risks and environmental exposures. Xenobiotics may be present in unheated and unfiltered methamphetamine injection preparations, and numerous impurities may be present in methamphetamine including carcinogens such as benzene [70,71,72]. Unsterile syringes and injection preparations that have not been filtered nor are subject to heating may increase risks for abscesses, phlebitis, and/or talcosis. In this study, 22.5% of IPWIDs self-reported ever having an abscess, which is somewhat comparable to a study of 169 non-Indigenous PWIDs in San Francisco where 32% had an abscess confirmed by physical exam [73]. Abscesses and other skin and soft tissue infections can be deadly and are common risks of injection drug use [74]. Using injection equipment contaminated with bacteria increases the likelihood of abscess development [75]. Staphylococcus Aureus is the most common bacteria to cause abscesses in PWIDs [76] and may be mitigated by the use of injection drug use syringe filters [77]. Future research focused on the methamphetamine injection practices of IPWIDs may be informative to the development of more comprehensive harm reduction interventions for IPWIDs in the US.

This research contributes a methodological advance to the study of polysubstance use in the life histories of IPWIDs. The ongoing development of a quantitative instrument that is used to collect data on sequential phenomena among IPWIDs has been built on the assumption that local lifeways, knowledge systems, and Indigenous methods such as storytelling and remembering can be articulated with Western scientific ways of knowing [21]. An overarching goal in developing the LHC is to not interrupt IPWIDs as they explain and remember substance use in nonlinear ways. In this study, we compared the ASI adapted for use with American Indians and the LHC to evaluate the instruments’ ability to detect substances reported over the life course. While no statistically significant differences were observed in the detection of any lifetime substance use between the ASI and LHC, the explicit screen for methamphetamine use and Suboxone^®^ use were visually apparent to participants, which reflected greater years of reported use in the sample for methamphetamine and Suboxone^®^, which were generally injected. The greatest similarities were observed between the ASI and LHC were for non-injected drugs (inhalants, alcohol, and cannabis). Generally, the LHC required less data collector training than the ASI 5th Edition, which may be why more methamphetamine and diverted Suboxone^®^ use were detected by the LHC. On the ASI, the calculation of years of use for a substance was typically conducted by data collectors performing subtraction between the first and last years of use, whereas the LHC visually assisted participants with anchoring their responses to life events, thereby producing gap years in reported years of use for substances. Some questions on the ASI 5th Edition adapted for use with American Indians regarding sanctioned or traditional use of substances could produce the unintended consequence of shaming participants who do not endorse these items and should be carefully considered relative to participants’ tribal affiliations and traditional practices. Our use of LHCs combined with social sequence analysis elucidated crude affinity typologies between IPWIDs who shared similar experiences of polysubstance use that emerge through synchronous acts over the lifespan. Further methodological research on the operational cost regimes and methods of class identification may be informative to future studies of polysubstance use in this population.

### Limitations

This study has several limitations. First, the small sample size limits the generalizability of the findings and the ability to meaningfully detect a larger number of PUC clusters; however, the combined use of the LHC and social sequence analysis may be useful in other tribal communities with similar experiences of polysubstance use. While the number of PUCs could have been further reduced given the small sample size, we also recognize the value of illustrating more variability across the lifespan in the context of a pilot study. This study considered PUCs across the lifespan of IPWIDs, and the smallest increment of time measured by the LHC was one year. Sequences occurring within a given year could not be distinguished, nor could the concomitant use of ≥2 substances on a given occasion. This study did not include a priori biological tests for substances. These measures were not included in this pilot due to concerns about biospecimen collection among AI/ANs. Finally, the risk of differential misclassification with recall of substance use via the LHC is ubiquitous, as any cognitive impairment that may be related to prior substance use could affect recall of substances and years used.

## 5. Conclusions

In this pilot study of 40 Assiniboine and Sioux IPWIDs living in northeastern Montana, high rates of hepatitis C coupled with methamphetamine injection were detected across the lifespan. Across all polysubstance use combinations identified in the sample, an appreciably large increase in the use of stimulants occurred between the year prior to and following injection initiation. A three-cluster solution for sequences of polysubstance use across the last 20 years of life was identified, differentiating younger IPWIDs who were transitioning into polysubstance use involving stimulants and/or narcotic analgesics from adults engaged with narcotic analgesics and stimulants over longer periods of time, and adults most focused on stimulant use over time. Results underscore the need for locally accessible syringe exchange programs. Results inform an understanding of how engagement with psychostimulants across the lifespan may contribute to excess disease burdens for IPWIDs in the syndemic context and inform the development of strategic harm reduction and health promotion interventions focused on IPWIDs.

## Figures and Tables

**Table 1:** Demographic and background characteristics of study participants, *n* = 40.

Characteristic	*N* (%)
Gender identity	
Male	22 (55.0%)
Female	18 (45.0%)
Age	
20–30	9 (22.5%)
30–40	18 (45.0%)
40–50	11 (27.5%)
50–65	2 (5.0%)
Relationship status	
Single	22 (55.0%)
Divorced/separated	2 (5.0%)
Married	4 (10.0%)
Unmarried w/partner	10 (25.0%)
Widowed/widower	2 (5.0%)
Tribal recognition status	
Registered member of a federally recognized tribe	35 (87.5%)
Associate tribal member	5 (12.5%)
Tribal affiliation	
Assiniboine	4 (10.0%)
Sioux	24 (60.0%)
Both Assiniboine and Sioux	9 (22.5%)
Other *	3 (7.5%)

* Includes Chippewa, Innuit, and Potawatomi.

**Table 2:** Injection drug use and general health characteristics of participants, *n* = 40.

Characteristic	*N* (%)
Age at first injection event	
<18	8 (20.0%)
18–21	14 (35.0%)
22–27	9 (22.5%)
>27	9 (22.5%)
Person who taught participant to inject	
Romantic partner	7 (17.5%)
Parent	2 (5.0%)
Sibling	2 (5.0%)
Other family member	9 (22.5%)
Friend	10 (25.0%)
Acquaintence	4 (10.0%)
Other	6 (15.0%)
Reason first injected substances	
Curiosity	12 (30.0%)
Intensity of high	10 (25.0%)
Interpersonal networks/social influence	14 (35.0%)
Coping with stressful life event	4 (10.0%)
Ever taught someone else to inject substances	11 (27.5%)
Shares syringes with others	
Never	15 (37.5%)
Sometimes	20 (50.0%)
Always	5 (12.5%)
Uses filter as part of normal injection routine	22 (56.4%)
Ever used drugs as part of religious practice or spiritual ceremony	1 (2.5%)
Substance use sanctioned by tribal leaders or medicine person	1 (2.5%)
Substance use common practice in traditional ways	2 (5.0%)
Traditional Indian cultural practices have been helpful in achieving or maintaining abstinence	17 (42.5%)
Ever overdosed	9 (22.5%)
Ever treated for alcohol abuse	23 (57.5%)
Ever treated for drug abuse	19 (47.5%)
Ever prescribed MOUD by a provider	5 (12.5%)
Ever had an abscess	9 (22.5%)
Ever diagnosed with Hepatitis C	18 (45%)

**Table 3:** Comparison of lifetime recall of substances used via the Life History Calendar and the Addiction Severity Index 5th Edition adapted for use with American Indians among Assiniboine and Sioux people who use injection drugs, *n* = 40.

	ASI 5th Edition Adapted for Use with American Indians		LHC			
Substance	No. Detected	Years of Reported Use ^a^	No. Detected	Years of Reported Use ^a^	McNemar’s Chi-Square	Pearson’s Correlation Coefficient
	N (%)	Mean (SD)	N (%)	Mean (SD)		
CNS Depressants						
Alcohol	40 (100%)	19.7 (9.5)	40 (100%)	21.2 (9.5)	--	0.82 ^b^
Sedatives/Hypnotics/Tranquilizers	6 (15.0%)	6.2 (6.2)	9 (22.5%)	3.8 (4.8)	1.29	0.40
Barbiturates	4 (10.0%)	8.5 (10.5)	1 (2.5%)	3.0 (--)	3.00	−0.41
CNS Stimulants						
Cocaine	21 (52.5%)	3.6 (5.2)	21 (52.5%)	5.7 (7.8)	0.00	0.19
Amphetamines	23 (57.5%)	12.7 (8.2)	15 (37.5%)	8.1 (9.7)	5.40	0.50
Methamphetamines	0 (0.0%)	0 (0.0%)	38 (95.0%)	14.8 (7.7)	--	--
Hallucinogens	20 (50.0%)	3.3 (5.2)	20 (50.0%)	2.2 (2.1)	0.00	0.17
Narcotic Analgesics						
Heroin	12 (30.0%)	2.1 (1.6)	13 (32.5%)	1.8 (1.3)	1.00	0.71
Suboxone	0 (0.0%)	0 (0.0%)	17 (42.5%)	1.5 (1.5)	--	--
Methadone	11 (27.5%)	3.1 (3.7)	8 (20.0%)	3.0 (2.2)	3.00	0.01
Other opiates/analgesics	25 (62.5%)	8.7 (8.1)	28 (70.0%)	5.7 (6.2)	3.00	0.45
Inhalants	15 (37.5%)	4.3 (8.2)	14 (35.0%)	2.9 (3.3)	0.33	0.84 ^b^
Cannabis	39 (97.5%)	22.2 (11.2)	38 (95.0%)	22.4 (10.8)	1.00	0.68 ^b^

Abbreviations: ASI Addiction Severity Index; LHC Life History Calendar; CNS Central Nervous System; SD Standard Deviation; P probability value. ^a^ Among participants reporting any use. ^b^ Statistically significant after Bonferroni–Holm correction procedure.

## Data Availability

The data presented in this study are available on request from the corresponding author. The data are not publicly available due to the sensitive nature of the data.

## Supplementary Materials

The following supporting information can be downloaded at: https://www.mdpi.com/article/10.3390/ijerph20010543/s1, File S1: Sequence index plots for each of the polysubstance use sequence classes identified
