# Supplementary material for: A Pilot Study of Polysubstance Use Sequences across the Lifespan among Assiniboine and Sioux People Who Use Injection Drugs
Source: Int J Environ Res Public Health. 2022 Dec 29;20(1):543. doi: 10.3390/ijerph20010543 (PMC9819103; doi:10.3390/ijerph20010543)
Supplement: Supplementary file 1 [file ijerph-20-00543-s001.zip › ijerph-2008235-SI.pdf]

Supplementary Material

Sequence index plots are shown below for each of the polysubstance use sequence classes identified in a sample of Assiniboine and Sioux people who use injection drugs (n=40). Polysubstance use combinations are shown relative to the 20 years of life preceding the interview. Age at time of polysubstance use combination is plotted on the X-axis. The number of individuals in the cluster is shown on the Y-axis, with individual bars (polysubstance use sequences) shown for each individual.

Cluster 1. (n=10)

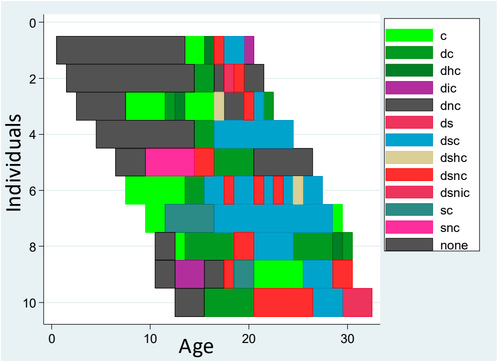

Cluster 2. (n=16)

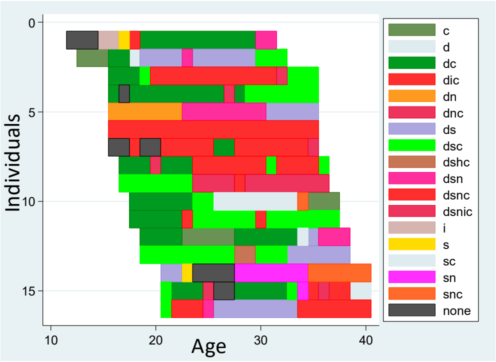

Cluster 3. (n=14)

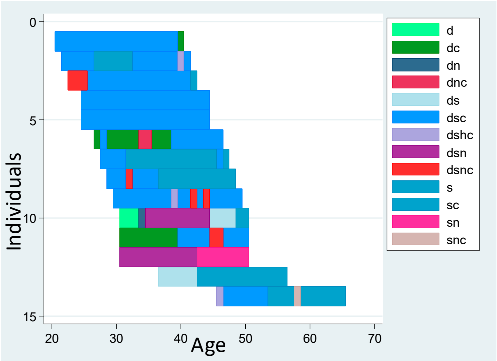

Legend

c	Cannabis
d	Depressants
h	Hallucinogens
n	Narcotic Analgesics
s	Stimulants
i	Inhalants
none	No substances used
